# Correction: Pediatric snakebite in Sub-Saharan Africa: Clinical predictors, outcomes, and gaps in care—A systematic review

**DOI:** 10.1371/journal.pntd.0014681

**Published:** 2026-08-28

**Authors:** Samuel Mayeden, Margit Wirth, Nisreen Agbaria, Masood Ali Shaikh, Peter Dambach, Michael Lowery Wilson, Olaf Horstick, Salvador Camacho, Hans-Jörg Lang, Andreas Deckert

The fourth author's initials appear incorrectly in the citation. The correct citation is: Mayeden S, Wirth M, Agbaria N, Shaikh MA, Dambach P, Lowery Wilson M, et al. (2026) Pediatric snakebite in Sub-Saharan Africa: Clinical predictors, outcomes, and gaps in care—A systematic review. PLoS Negl Trop Dis 20(2): e0013450. https://doi.org/10.1371/journal.pntd.0013450.
