# Supplementary material for: Pediatric snakebite in Sub-Saharan Africa: Clinical predictors, outcomes, and gaps in care—A systematic review
Source: PLoS Negl Trop Dis. 2026 Feb 19;20(2):e0013450. doi: 10.1371/journal.pntd.0013450 (PMC12945311; doi:10.1371/journal.pntd.0013450)
Supplement: S1 Table — Full electronic search strings used across all databases for identification of pediatric snakebite envenoming studies in sub-Saharan Africa. (DOCX) [file pntd.0013450.s001.docx]

**S1_Table Search Strategies for All Databases**

| Database | Search Strategy/Terms | Notes |
| --- | --- | --- |
| PubMed | ("Africa South of the Sahara" OR "sub-Sahara Africa" OR "sub-Saharan Africa" OR "central Africa" OR "East Africa" OR "eastern Africa" OR "South Africa" OR "Southern Africa" OR "West Africa" OR "Western Africa" OR Angola OR Benin OR Botswana OR Burkina Faso OR Burundi OR "Cabo Verde" OR Cameroon OR "Central African Republic" OR Chad OR Comoros OR Congo OR "Cote d´Ivoire" OR Djibouti OR "Equatorial Guinea" OR Eritrea OR Eswatini OR Ethiopia OR Gabon OR Gambia OR Ghana OR Guinea OR Guinea-Bissau OR "Ivory Coast" OR Kenya OR Lesotho OR Liberia OR Madagascar OR Malawi OR Mali OR Mauritius OR Mozambique OR Namibia OR Niger OR Nigeria OR Rwanda OR "Sao Tome and Principe" OR Senegal OR Seychelles OR Sierra Leone OR Somalia OR "South Sudan" OR Sudan OR Swaziland OR Tanzania OR Togo OR Uganda OR Zambia OR Zimbabwe OR "Africa South of the Sahara"[Mesh]) AND (infants, newborn OR infant OR child, preschool OR child OR adolescent OR pediatric) AND (snakebite OR "snake bite" OR "snake envenom*" OR "snake venom poisoning").  There were no filters applied. | No filters applied. Full search string in methods. |
| SafetyLit | The advanced search function was used and for all searches the “TEXTWORT + SYNONYM” search was applied. There were no search limits used. The country-related block of the search string was combined with the children-related block and the snakebite-related block with “AND”. There were only 6 fields for every block available, which was sufficient for the infant and snakebite-related block of the search string, but not for the country-related, so 8 separated searches had to be done, one for 6 countries simultaneously.  The search strategy was the following: children OR infant OR adolescent OR paediatric AND snakebite OR snake bite OR snake envenomation OR snake envenoming OR snake venom poisoning AND  Search 1: Africa OR Angola OR Benin OR Botswana OR Burkina Faso OR Burundi  Search 2: Cabo Verde OR Cameroon OR Central African Republic OR Chad OR Comoros OR Congo  Search 3: Côte d'Ivoire OR Djibouti OR Equatorial Guinea OR Eritrea OR Eswatini OR Ethiopia  Search 4: Gabon OR Gambia OR Ghana OR Guinea OR Guinea-Bissau OR Ivory Coast  Search 5: Kenya OR Lesotho OR Liberia OR Madagascar OR Malawi OR Mali  Search 6: Mauritius OR Mozambique OR Namibia OR Niger OR Nigeria OR Rwanda  Search 7: Sao Tome and Principe OR Senegal OR Seychelles OR Sierra Leone OR Somalia OR Sudan  Search 8: Swaziland OR Tanzania OR Togo OR Uganda OR Zambia OR Zimbabwe | Advanced search, split due to platform limits; results combined from all searches. |
| Google Scholar | The search had to be divided into 6 separate searches again due to the length of the search box. Every one of the searches included:  (infants OR children OR adolescent OR pediatric)  AND  (snakebite OR "snake bite" OR "snake envenomation" OR "snake envenoming" OR "snake venom poisoning")  AND  Search 1: (Africa OR Angola OR Benin OR Botswana OR Burkina Faso OR Burundi OR "Cabo Verde" OR Cameroon)  Search 2: ("Central African Republic" OR Chad OR Comoros OR Congo OR "Cote d´Ivoire" OR Djibouti)  Search 3: (Eritrea OR Eswatini OR Ethiopia OR Gabon OR Gambia OR Ghana OR Guinea OR "Ivory Coast" OR Kenya)  Search 4: (Lesotho OR Liberia OR Madagascar OR Malawi OR Mali OR Mauritius OR Mozambique OR Namibia OR Niger)  Search 5: (Nigeria OR Rwanda OR "Sao Tome and Principe" OR Senegal OR Seychelles OR Sierra Leone OR Somalia)  Search 6: (Sudan OR Swaziland OR Tanzania OR Togo OR Uganda OR Zambia OR Zimbabwe)  The search results were sorted by relevance and citations were included in the search results. No other filters were applied. For every search, the first 50 hits were screened by title and abstract. If there appeared no new hits eligible for full text screening among the last 20 results, the search was stopped. If there were still new relevant hits, the next 50 hits underwent title and abstract screening. This proceeding continued until there were no more new eligible results. | Search split by character limit. First 50 results screened by relevance. |
| African Journals Online | The search box was also too short for a longer search string. The keywords “snakebite Africa children” were used to search the database. The hits were sorted by relevance. Only the first 100 hits were accessible. | Short phrase due to platform limits. First 100 hits screened by relevance. |
| CINAHL | The advanced search option was used. “TX all text” search and “find all my search terms” search mode was chosen; the only applied limitation was “Scholarly (Peer Reviewed) Journals”.  infant or newborn or child or adolescent or pediatric  AND  snakebite or snake bite or snake venom poisoning or snake envenomation or snake envenoming  AND  Africa South of the Sahara OR sub-Sahara Africa OR sub-Saharan Africa OR central Africa OR East Africa OR eastern Africa OR South Africa OR Southern Africa OR West Africa OR Western Africa OR Angola OR Benin OR Botswana OR Burkina Faso OR Burundi OR Cabo Verde OR Cameroon OR Central African Republic OR Chad OR Comoros OR Congo OR Cote d´Ivoire OR Djibouti OR Equatorial Guinea OR Eritrea OR Eswatini OR Ethiopia OR Gabon OR Gambia OR Ghana OR Guinea OR Guinea-Bissau OR Ivory Coast OR Kenya OR Lesotho OR Liberia OR Madagascar OR Malawi OR Mali OR Mauritius OR Mozambique OR Namibia OR Niger OR Nigeria OR Rwanda OR Sao Tome and Principe OR Senegal OR Seychelles OR Sierra Leone OR Somalia OR South Sudan OR Sudan OR Swaziland OR Tanzania OR Togo OR Uganda OR Zambia OR Zimbabwe | Limit: Peer Reviewed Journals. Full search string in methods. |
