# Supplementary material for: Pediatric snakebite in Sub-Saharan Africa: Clinical predictors, outcomes, and gaps in care—A systematic review
Source: PLoS Negl Trop Dis. 2026 Feb 19;20(2):e0013450. doi: 10.1371/journal.pntd.0013450 (PMC12945311; doi:10.1371/journal.pntd.0013450)
Supplement: S2 Table — Summary of extracted study characteristics, including country, study design, sample size, key clinical features, management, and main outcomes reported in included pediatric snakebite studies. (DOCX) [file pntd.0013450.s002.docx]

**S2_Table Characteristics and Key Findings of Included Studies on Pediatric Snakebite in Sub-Saharan Africa**

| Ref No.. | First Author (Year) | Country | Study Design | Sample Size (n; ediatric/all) | Age Range (years) | Circumstance  /Seasonality | Time to Hospital | M: F Ratio | Traditional  /First Aid | Antivenom Use (%; Adverse Reactions) | Prognostic Factors Identified | Clinical Presentation  /Complications | Outcomes |
| --- | --- | --- | --- | --- | --- | --- | --- | --- | --- | --- | --- | --- | --- |
| [1] | Abdullahi et al. (2022) | Ethiopia | Cross-sectional | 430/170 | <10, 10–25, 26–40, 41–55, >55 | Higher risk at rainy season (Apr–Jun, Oct–Dec); Daytime indoors: 85 Daytime outdoors: 155 Nighttime indoors: 140 Nighttime outdoors: 50 Walking in bush: 220 Cleaning garbage: 40 Sleeping in home: 120 Trying to kill snake: 50 | Within 1h: 30 2–3h: 20 3–4h: 10 4–6h: 30 >6h: 340 | 255/175 | Herbal: 100 Tourniquet: 60 None: 270 | Not administered | Age <10y: AOR 4.63 (2.13–11.38) Age 10–30y: AOR 2.42 (1.39–9.30) Urban: AOR 0.56 (0.06–0.97) Daytime: AOR 0.49 (0.31–0.87) Bite-to-hospital >6h: AOR 2.01 (1.39–4.05) | Fang marks: 65 Pain: 105 Swelling: 135 Necrosis: 5 Neuro: 15 Bleeding: 15 Anaphylaxis/hemo instability: 20 No systemic: 380 Amputation: 5 Tissue loss: 90 | Death: 20 (4.7%) Limb amputation: 5 (1.2%) Referred: 20 (4.7%) Improved/discharged: 295 (68.6%) Local tissue loss: 90 (20.9%) |
| [2] | Buitendag et. al (2021) | South Africa | Case series | 274/274 | Up to 13 y | Peak incidence Dec–Feb | Median 7 h (range 0.5–79 h) | 56% / 44% | Not reported | Applied in 53/274; 23% of cytotoxic, 100% haemotoxic/neurotoxic; 25 adverse reactions | Delay in presentation (and thus antivenom) was the most significant risk for limb amputation; no correlation between age and ICU or hypersensitivity | 71% cytotoxic, 2% haemotoxic, 1% neurotoxic, 26% no envenomation. AKI: 6 (5 cytotoxic, 6 haemotoxic); 56 pts (20%) had procedures (32 graft, 54 debride, 2 fasciotomy, 5 BKA). Cytotoxic group: majority of procedures. Adverse reactions to antivenom: 25. | Mortalities: 0; Amputation: 5 BKA (1.8%); Procedures: 56 (20%) |
| [3] | Kasturiratne et al. (2008) | South Africa & neighbors | Cross-sectional | 2553/412 <11y | 0–1, 2–4, 5–9, 10–14, 15–19, ... 60+ | Peak warm months (Jan–Mar); hand bites (42%) in young White males handling snakes | Not reported | 3:1 White, 55% M / 45% F Black | Not reported | All patients treated with antivenom; only 2% received ≥50 ml, 65% only 10 ml (1 ampoule); 25% wrong indication; serum sensitivity 10 (0.3%) | Serious bites and death rate higher in <11y (death rate 5.1% vs 1.4% in older); hand bites in White males | Bite location: 72.7% lower limb, 23.6% hand/arm, 2% trunk/neck/head; 19% had no symptoms except pain; mortality: 2%, higher in <11y (5.1%) | 21 deaths among 412 children <11y (5.1%); overall mortality 2%; wrong antivenom indication ~25% cases |
| [4] | Habib et al., 2015 | Gambia | Case series | 28/28 | 4–12 | Rainy (16, 57%), dry (12, 43%); 6 bites at home, 3 farm, 1 school; Morning: 6, Afternoon: 4, Evening: 5, Night: 4 | Mean 10.3 h | 2:1 | 6 (21%) tourniquets | 6/28 (21%) received antivenom; 3/6 died | Shock, restlessness, regional adenitis linked to poor prognosis | Localization: leg 8, arm 5; Swelling (>50% limb) 20, shock 5, restlessness 4, adenopathy 4, bleeding 3, ulceration 2, fever 2, vomiting 2, blisters 2, pruritus 1, convulsions 1, compartment 2; Antibiotics 15, tetanus 7, fasciotomy 1 | Mortalities: 4 (14.3%); Mean hospital stay: 3.63 d; 3/6 died after antivenom |
| [5] | Hadly, T. et al. (1999) | South Africa | Diagnostic study | 51/51 | Only children, age not specified | Not reported | Not reported | Not reported | 18/44 had tourniquet | Restricted to severe/progressive cases; % NR | Sensitivity of TEG for severe course: 94%; specificity: 46%. Normal TEG PPV for mild course: 94%. INR not significant (p=0.2). Both normal: 86% PPV mild; both abnormal: 70% PPV severe | 35/51 had abnormal TEG. 1 with normal TEG on admission later developed severe diathesis (TEG became abnormal). 17/35 with abnormal TEG developed severe picture (p=0.003). No significant INR effect. | LOS: abnormal TEG mean 5.6 d, normal TEG mean 2.5 d. Antivenom only in severe cases. |
| [6] | Hernández et al. (2019) | South Africa | Multivariable prediction model | 72/72 | 6 mo – 15 y | Not reported | Median 20 h [IQR 11–31]; 35% >24h | 60% M | Not reported | 9/72 (12.5%) required antivenom; 2/9 (22%) anaphylaxis (ICU) | Fasciotomy risk: Higher ISS, tachycardia, high shock index (most predictive), leukocytosis, Hb <11, INR >1.2, delay >24h (83% vs. 27.7%, p=0.04). Antivenom need: lower SBP, lower Hb, more severe symptoms; complications higher with antivenom (66% vs. 30%, p=0.04). | Location: foot 38%, leg 25%, arm 21%, hand 12.5%, head/neck 3.5%. Syndromes: PPS 88%, weakness 7%, bleeding 5%. 49% high shock index; 10 ICU; median stay 5d [2–8]. Complications: 25 total (skin graft 7%, cellulitis 5.5%, abscess 4.2%, contracture 4.2%, anaphylaxis 2.8%, compartment 2.8%). Readmissions: 8. | Mortalities: 0. Fasciotomy: 18 (25%). Median stay longer for antivenom (11d [6–18] vs 4d [2–7], p=0.03). Complications: 66% with antivenom vs 30% without. |
| [7] | Ndu et al. (2018) | Nigeria | Case series | 13/13 | 7–17 | 46.2% urban, 15.4% semiurban, 38.5% rural; 84.6% rainy season (Apr–Oct); 84.6% at home; 84.6% at night | <2 h: 4 (30.8%), >2 h: 9 (69.2%) | 1:1.2 | Local incision: 77%, tourniquet: 69%, black stone: 46%, herbs: 54%, native doctor: 54% | 8/13 (61.5%) received antivenom; 3/8 (37.5%) allergic reaction | Late presentation (≥2 h) significantly linked to longer hospital stay | Lower limb 69%. Pain 100%, swelling 77%, fever 69%, bleeding 39%. No ptosis/resp distress/shock. Severity: none 3, mild 1, moderate 5, severe 4. Treatment: analgesia 100%, antibiotics 92%, tetanus 77%, antivenom 62%, transfusion 23%, fasciotomy 8%. Complications 38.5% (1 gangrene, 1 ulcer, 3 compartment). | 9 (69%) recovered/discharged, 3 (23%) left AMA, 1 death (7.7%). Mean hospital stay: 3.2 d (range 1–11). |
| [8] | Ilyasu et al. (2022) | Nigeria | Prospective cohort | 128/28 (1–10 y) | 1–10, 11–20, 21–30, ... 51–60 | 41% farming, 12% herding | median 16.0 h (range 1–237 h) | 79% M, 21% F | Not reported | Given for systemic envenomation; repeated as needed; provided free by govt; dose: 10–30 mL; anaphylaxis managed per protocol | Blister predicted poor outcome (OR=0.33, 95%CI 0.11–0.96, p=0.04); blisters linked to higher gangrene (9.5% vs 1.2%), non-clotting (95% vs 70%), higher antivenom need; no mortality diff by group | Most common: lower limb 68%. Pain 68%, swelling 98%, blister 33%. Local bleeding 66%, systemic bleeding 26%. Non-clotting 78%, gangrene 4%, amputation 2%, LOC 2%, death 3%. Poor outcome (blister) 33%. | 4/128 deaths (3.1%); 3/128 amputation (2.3%). Death in cobra 20%, carpet viper 2.2%, none in night adder. Median stay: with blister 6d, no blister 5d. 42/128 poor outcome. |
| [9] | Einterz et al. (2003) | Cameroon | Case series | 48/15 (<16y) | 8 mo–53 y; 0–5, 6–10, 11–15, ... | Not reported | Average 14 h | 1.3:1 | Not reported | None | Severe clinical picture in 80% of children <11y. Early hemorrhagic shock only in children (3/48). Edema > root of limb: high risk (81% vs 12.5%). Tachycardia or fever >48h = complications. Hb <9g/100ml at day 3 = poor prognosis, >11g/100ml favorable. | Echis carinatus only. Mortality 36.4% (17/48). 0–5y: all 4 died; 6–10y: 4/6 died; 11–15y: all 5 survived. Important swelling in 16/48. Prolonged bleeding >24h in 39.5%. Hemorrhagic signs 64.6%. Average coagulopathy 3.7 d (6.25 d in fatal, 1.95 d in others). | 17 deaths (36.4%). High mortality in younger ages. No antivenom given. |
| [10] | Wood et.al. (2009) | South Africa | Case series | 206/99 | <17y, 17y+ | Not reported | Not reported | 1.8:1 children, 0.9:1 adult | Not reported | Not reported | Children <17y: higher need for surgery and prolonged hospital stay (>7d) than adults | Compartment syndrome in 9 children (all lower limb). 6/99 children (6%) needed surgery vs 1/107 adults (0.9%). 2 above-knee amputations in children after compartment syndrome. 3 needed fasciotomies/split-skin grafts. | 33/99 children (33%) had hospital stay >7 days vs 14/107 adults (13%, p<0.01). No deaths reported. |
| [11] | Steegemans et al. (2022) | Ethiopia | Cohort study | 250/57 (<18y) | <15y, 15–45y, >45y | Peak Sep–Nov; 77% field; 23% home; 82% daytime, 18% night | <12h: 54 (26.3%), >12h: 151 (73.7%) | 81% M, 19% F | Tourniquet 16%, cultural med 7% | 162/207 (79%) antivenom; 6/162 (3.7%) adverse reactions | Rural residence (AOR=2.52), bacterial superinfection (AOR=4.69), clinical stage 3/4 (AOR=4.84), no antivenom (AOR=6.65) | Upper extremity 15%, lower extremity 85%. Clinical stage: 1 (27.5%), 2 (65%), 3 (5%), 4 (2.5%). Fever 45%, swelling 65%, bleeding 84%, bacterial superinfection 63%. No neurotoxic symptoms. Anemia 52%, thrombocytopenia 26%, leukocytosis 33%, prolonged clotting 20%. | Discharged/improved 72%, death 11%, disability/amputation 0.5%, referred/left AMA 17%. Median length of stay not reported. |
| [12] | Nduagubam et al. (2020) | Nigeria | Case series | 25/17 | Not reported | 40% farm, 32% home, 28% walking. May–Aug peak. Daytime 65%. | <6h: 32%, 6–12h: 8%, 12–24h: 8%, >24h: 52%. Range: 0.5–168h | 1.4:1 | 48% traditional: concoctions, tourniquet, local incision. All late arrivals had used traditional practices. | 23/25 (92%) antivenom; 3/23 (13%) adverse reactions. | Late presentation: longer WBCT. Not associated with more adverse anti-venom effects. | Lower limb 72%, upper 24%, neck 4%. Pain/swelling 76%, bleeding 52%. 8 (32%) had complications: dysphagia, hematemesis, hematuria, neck stiffness, abdominal distension, seizure, neck swelling. All complications were late presenters. | 76% discharged, 16% left against medical advice, 4% absconded, 4% died (death after late antivenom due to funds). |
| [13] | Sloan et al. (2007) | South Africa | Mixed methods, case series | 694/146 (<10y) | <10y, <20y | 70% Nov–Mar; 78% walking; 14% sleeping; rest work/toilet; 94% barefoot | 60% >6h; median 7h15min | 47% M, 53% F | 80% imbibed isibiba (oral traditional), 35% scarification, 37.5% self-medicated; delays greatest after THP consultation | 10% received antivenom; median time from bite to antivenom: 7 h (range 5–10h) | THP consultation linked to longer delays; PHC visit and age >9y trend to delay; self-admin not delayed but linked to severity | 88% bites to foot, 12% upper limb. Median hospital stay 3d. No fatalities. 10% received antivenom (median 7h after bite, range 5–10h) | No deaths. Median hospital stay 3d. |
| [14] | Variawa S, et al. (2021) | South Africa | Case series | 51/51 | <14y | Not reported | Median 10h (range 10min–50h); mild 6h, mod 7h, severe 12h | 57% M, 43% F | Not reported | 46% (23/51) antivenom; 13% allergic, 57% anaphylaxis | No correlation age/gender & severity/AKI; INR correlates with severity; WCC/renal fxn do not | Cytotoxic only. 18% mild, 47% moderate, 35% severe. 22% AKI (1 mild, 7 mod, 3 severe). Procedures: 1 fasciotomy, 15 debridement, 3 skin graft. 1 (2%) death. No amputations. | 1 death (2%). 2 intubated/ventilated. 1 CPR. Adverse drug reactions in 70%: 3/23 allergic, 13/23 anaphylaxis. Higher risk of anaphylaxis in children suggested. |
| [15] | Wood et al. (2016) | South Africa | Case series | 879/222 (<12y) | 0–5y, 6–10y, 11–15y, ... 80–85y | Peak Dec–Mar | Median 5 h (IQR 3–10h, n=743). Moderate: 5h, Severe: 10h | 1:1 | Not reported | 96/879 (11.5%) received antivenom; 23/96 (23%) anaphylaxis | <12y vs older (OR 3.26, CI 2.19–4.87), INR>1.5 (OR 2.25), PLT<100 (OR 2.35), Hb<8 (OR 5.68), WCC>10 (OR 3.15); delay increased severity | 74% <12y envenomed vs 57% >12y. Severe PPS 16%. Isolated haemotoxic/neurotoxic rare. Surgery for PPS (16%); most common: debridement, skin graft. Amputation 6%. No dialysis. | No deaths. Severe PPS/surgery more frequent in children. Children twice as likely to require antivenom. Median antivenom dose 40ml (IQR 40–100). |
| [16] | Wood et al. (2016) | South Africa | Case series | 42/25 (<12y) | <6y, <12y, 12y+ | Not reported | Not reported | 1.3:1 | Not reported | 18 received polyvalent antivenom | No increased risk of severe intramuscular involvement in children; assessed swelling by ultrasound (foot 12, leg 3, hand 12, forearm 13, upper arm 2). Subcutaneous expansion > muscle; expansion coefficients: foot 1.3, hand 1.5, upper arm 1.8, forearm 1.9, leg 3.3; complications: 1 fasciotomy, 16 debridements | No correlation between expansion coefficients and need for antivenom or debridement. |  |
| [17] | Wood et al. (2017) | South Africa | Multivariable prediction model study | Dev: 879/220 (<12y); Val: 100/57 (<12y) | <12y, 12+y | Not reported | Validation: mean 9.5h (SD 17.4h), 47% <6h, 53% ≥6h | Not reported | Not reported | ATI predictors: age <14y, duration to admission >7h, WCC >10x10^9/L, INR >1.2, Platelets <92x10^9/L, Hb <7.1g/dL (each 1 pt); 4+ pts = ATI | 879 in dev: 146 received ATI (antivenom 64, surgery 49, both 33); Val: antivenom 19, surgery 4, both 17 | Antivenom use: Dev: 64/879 antivenom alone, 33 both; Val: 19/100 antivenom, 17 both. |  |
| [18] | Abouyannis et al. (2023) | Kenya | Case series / Prediction model | 584/584 | ≤12y (<1y, 1y, 2–5y, 6–9y, 10–12y) | Outdoor/near home 42.7%, house 22.8%, footpath 16.6%, farming 9.8%, bush 4.6%, school 2.0%, hunting 1.6% | Median 6h45min (IQR 3–15h; 10min–17d). Traditional therapy: median 9.4h, no traditional: 5.8h (p=0.003) | 52.4% male, 47.6% female | Black stone: 23.3%, tourniquet: 10.2%, cutting: 10.0%, herbal: 8.9%, suction: 0.6%, ≥2 methods: 11.9% | 119/472 (25.2%) received antivenom (most for local envenoming); By age: 0–5y: 33.9%, 6–12y: 22%; 1 vial: 67.9%, 2 vials: 24.5%, ≥3 vials: 7.5%; Allergic rxn: 17.6%, anaphylaxis: 0.8% | Predictors for severe local tissue damage: upper limb bite (OR 3.27), WCC (OR 1.14), SBP (OR 1.03), Na (OR 0.9), Hb (OR 0.72) | Localization: foot 52.2%, leg 33.5%, hand 10.2%, arm 3.6%; swelling 84.5%, blisters 8.5%, severe local tissue damage/necrosis 3.8%, required surgery 3.3%, amputation 0.5%, mortality 0.9% | Discharged improved: majority; Mortality: 5 (0.9%) |

**Abbreviations:** *PPS = Painful Progressive Swelling; INR = International Normalized Ratio; PLT = Platelet Count; Hb = Hemoglobin; WCC = White Cell Count; AKI = Acute Kidney Injury; CPR = Cardiopulmonary Resuscitation; THP = Traditional Health Practitioner; PHC = Primary Health Clinic; AOR = Adjusted Odds Ratio; Neuro = Neurologic Disorders; Hemo = Hemodynamic; BKA = Below Knee Amputation; ICU = Intensive Care Unit; M = Male; F = Female; TEG = Thrombelastogram; PPV = Positive Predictive Value; LOS = Length of Stay; ISS = Injury Severity Score; SBP = Systolic Blood Pressure; LOC = Loss of Consciousness; OR = Odds Ratio; AMA = Against Medical Advice; WBCT = Whole Blood Clotting Time.*

**References**

1. Abdullahi, A., Yusuf, N., Debella, A., Eyeberu, A., Deressa, A., Bekele, H., Ketema, I., Abdulahi, I. M., & Weldegebreal, F. (2022). Seasonal variation, treatment outcome, and its associated factors among the snakebite patients in Somali region, Ethiopia. *Frontiers in Public Health*, *10*, 901414. <https://doi.org/10.3389/fpubh.2022.901414>
2. Buitendag J, Variawa S, Wood D, Oosthuizen G. An analysis of paediatric snakebites in north-eastern South Africa. S Afr j surg. 2021;59. doi:[10.17159/2078-5151/2021/v59n3a3500](https://doi.org/10.17159/2078-5151/2021/v59n3a3500)
3. Kasturiratne, A., Wickremasinghe, A. R., De Silva, N., Gunawardena, N. K., Pathmeswaran, A., Premaratna, R., Savioli, L., Lalloo, D. G., & De Silva, H. J. (2008). The global burden of snakebite: A literature analysis and modelling based on regional estimates of envenoming and deaths. PLoS Medicine, 5(11), e218. <https://doi.org/10.1371/journal.pmed.0050218>
4. Habib, A. G., Kuznik, A., Hamza, M., Abdullahi, M. I., Chedi, B. A., Chippaux, J.-P., & Warrell, D. A. (2015). Snakebite is under appreciated: Appraisal of burden from west africa. *PLOS Neglected Tropical Diseases*, *9*(9), e0004088. <https://doi.org/10.1371/journal.pntd.0004088>
5. Hadley GP, McGarr P, Mars M. The role of thromboelastography in the management of children with snake-bite in southern Africa. Transactions of the Royal Society of Tropical Medicine and Hygiene. 1999;93: 177–179. doi:[10.1016/S0035-9203(99)90300-0](https://doi.org/10.1016/S0035-9203(99)90300-0)
6. Hernandez MC, Traynor M, Bruce JL, Bekker W, Laing GL, Aho JM, et al. Surgical Considerations for Pediatric Snake Bites in Low‐ and Middle‐Income Countries. World j surg. 2019;43: 1636–1643. doi:[10.1007/s00268-019-04953-9](https://doi.org/10.1007/s00268-019-04953-9)
7. Ndu, I., Edelu, B., & Ekwochi, U. (2018). Snakebites in a Nigerian children Population: A 5-year review. *Sahel Medical Journal*, *21*(4), 204. <https://doi.org/10.4103/smj.smj_18_18>
8. Iliyasu, G., Dayyab, F. M., Michael, G. C., Hamza, M., Habib, M. A., Gutiérrez, J. M., & Habib, A. G. (2023). Case fatality rate and burden of snakebite envenoming in children – A systematic review and meta-analysis. Toxicon, 234, 107299. <https://doi.org/10.1016/j.toxicon.2023.107299>
9. Einterz EM, Bates ME. Snakebite in northern Cameroon: 134 victims of bites by the saw-scaled or carpet viper, Echis ocellatus. Trans R Soc Trop Med Hyg. 2003;97: 693–696. doi:[10.1016/s0035-9203(03)80105-0](https://doi.org/10.1016/s0035-9203(03)80105-0)
10. Wood D, Webb C, DeMeyer J. Severe snakebites in northern KwaZulu-Natal: treatment modalities and outcomes. S Afr Med J. 2009;99: 814–818.
11. Steegemans I, Sisay K, Nshimiyimana E, Gebrewold G, Piening T, Menberu Tessema E, et al. Treatment outcomes among snakebite patients in north-west Ethiopia—A retrospective analysis. Ainsworth SR, editor. PLoS Negl Trop Dis. 2022;16: e0010148. doi:[10.1371/journal.pntd.0010148](https://doi.org/10.1371/journal.pntd.0010148)
12. Nduagubam, O. C., Chime, O. H., Ndu, I. K., Bisi-Onyemaechi, A., Eke, C. B., Amadi, O. F., & Igbokwe, O. O. (2020). Snakebite in children in Nigeria: A comparison of the first aid treatment measures with the world health organization’s guidelines for management of snakebite in Africa. *Annals of African Medicine*, *19*(3), 182–187. <https://doi.org/10.4103/aam.aam_38_19>
13. Sloan, D. J., Dedicoat, M. J., & Lalloo, D. G. (2007). Healthcare‐seeking behaviour and use of traditional healers after snakebite in Hlabisa sub‐district, KwaZulu Natal. Tropical Medicine & International Health, 12(11), 1386–1390. <https://doi.org/10.1111/j.1365-3156.2007.01924.x>
14. Variawa S, Buitendag J, Marais R, Wood D, Oosthuizen G. Prospective review of cytotoxic snakebite envenomation in a paediatric population. Toxicon. 2021;190: 73–78. doi:[10.1016/j.toxicon.2020.12.009](https://doi.org/10.1016/j.toxicon.2020.12.009)
15. Wood, D., Sartorius, B., & Hift, R. (2016). Classifying snakebite in South Africa: Validating a scoring system. *South African Medical Journal*, *107*(1), 46. <https://doi.org/10.7196/SAMJ.2017.v107i1.11361>
16. Wood, D., Sartorius, B., & Hift, R. (2016). Snakebite in north-eastern South Africa: Clinical characteristics and risks for severity. *South African Family Practice*, *58*(2), 62–67. <https://doi.org/10.1080/20786190.2015.1120934>
17. Wood D, Webb C, DeMeyer J. Severe snakebites in northern KwaZulu-Natal: treatment modalities and outcomes. S Afr Med J. 2009;99: 814–818.
18. Abouyannis, M., Boga, M., Amadi, D., Ouma, N., Nyaguara, A., Mturi, N., Berkley, J. A., Adetifa, I. M., Casewell, N. R., Lalloo, D. G., & Hamaluba, M. (2023). A long-term observational study of paediatric snakebite in Kilifi County, south-east Kenya. PLOS Neglected Tropical Diseases, 17(7), e0010987. <https://doi.org/10.1371/journal.pntd.0010987>
