# Supplementary material for: Pediatric snakebite in Sub-Saharan Africa: Clinical predictors, outcomes, and gaps in care—A systematic review
Source: PLoS Negl Trop Dis. 2026 Feb 19;20(2):e0013450. doi: 10.1371/journal.pntd.0013450 (PMC12945311; doi:10.1371/journal.pntd.0013450)
Supplement: S3 Table — Methodological quality appraisal results for included studies using standardized assessment criteria, categorized as high, moderate, or low quality. (DOCX) [file pntd.0013450.s003.docx]

**S3_Table Quality Assessment Scores of Included Studies**

| **Ref. No** | **Study** | **Country** | **Quality Assessment Tool** | **Score** | **Max Score** | **Quality Rating** |
| --- | --- | --- | --- | --- | --- | --- |
| [1] | Abouyannis et al. (2023) | Kenya | STROBE (cross-sectional) | 21 | 24 | Moderate |
| [2] | Abdullahi et al. (2022) | Ethiopia | STROBE (cross-sectional) | 13 | 24 | Low |
| [3] | Kasturiratne et al. (2008) | South Africa | STROBE (cross-sectional) | 16 | 24 | Moderate |
| [4] | Habib et al., (2015) | Gambia | JBI Case Series | 8 | 10 | Moderate |
| [5] | Hadley (1999) | South Africa | STARD | 21 | 25 | High |
| [6] | Hernández et al. (2019) | South Africa | TRIPOD (development) | 20 | 22 | High |
| [7] | Ndu et al. (2018) | Nigeria | JBI Case Series | 8 | 10 | Moderate |
| [8] | Ilyasu et. al (2022) | Nigeria | STROBE (cohort) | 22 | 24 | High |
| [9] | Einterz et al. (2003) | Cameroon | JBI Case Series | 5 | 10 | Low |
| [10] | Buitendag et. al (2021) | South Africa | JBI Case Series | 9 | 10 | High |
| [11] | Steegemans et al. (2022) | Ethiopia | STROBE (cohort) | 27 | 30 | High |
| [12] | Nduagubam et al. (2020) | Nigeria | JBI Case Series | 10 | 10 | High |
| [13] | Sloan et al. (2007) | South Africa | JBI Case Series | 8 | 10 | Moderate |
| [14] | Variawa S., et al (2021) | South Africa | JBI Case Series | 10 | 10 | High |
| [15] | Wood et al. (2016a) | South Africa | JBI Case Series | 10 | 10 | High |
| [16] | Wood et al. (2016b) | South Africa | JBI Case Series | 10 | 10 | High |
| [17] | Wood et al. (2017) | South Africa | TRIPOD (dev. & validation) | 30 | 35 | High |
| [18] | Wood et. al. (2009) | South Africa | JBI Case Series | 10 | 10 | High |

**Reference**

1. Abouyannis, M., Boga, M., Amadi, D., Ouma, N., Nyaguara, A., Mturi, N., Berkley, J. A., Adetifa, I. M., Casewell, N. R., Lalloo, D. G., & Hamaluba, M. (2023). A long-term observational study of paediatric snakebite in Kilifi County, south-east Kenya. PLOS Neglected Tropical Diseases, 17(7), e0010987. <https://doi.org/10.1371/journal.pntd.0010987>
2. Abdullahi, A., Yusuf, N., Debella, A., Eyeberu, A., Deressa, A., Bekele, H., Ketema, I., Abdulahi, I. M., & Weldegebreal, F. (2022). Seasonal variation, treatment outcome, and its associated factors among the snakebite patients in Somali region, Ethiopia. *Frontiers in Public Health*, *10*, 901414. <https://doi.org/10.3389/fpubh.2022.901414>
3. Kasturiratne, A., Wickremasinghe, A. R., De Silva, N., Gunawardena, N. K., Pathmeswaran, A., Premaratna, R., Savioli, L., Lalloo, D. G., & De Silva, H. J. (2008). The global burden of snakebite: A literature analysis and modelling based on regional estimates of envenoming and deaths. PLoS Medicine, 5(11), e218. <https://doi.org/10.1371/journal.pmed.0050218>
4. Habib, A. G., Kuznik, A., Hamza, M., Abdullahi, M. I., Chedi, B. A., Chippaux, J.-P., & Warrell, D. A. (2015). Snakebite is under appreciated: Appraisal of burden from west africa. *PLOS Neglected Tropical Diseases*, *9*(9), e0004088. <https://doi.org/10.1371/journal.pntd.0004088>
5. Hadley GP, McGarr P, Mars M. The role of thromboelastography in the management of children with snake-bite in southern Africa. Transactions of the Royal Society of Tropical Medicine and Hygiene. 1999;93: 177–179. doi:[10.1016/S0035-9203(99)90300-0](https://doi.org/10.1016/S0035-9203(99)90300-0)
6. Hernandez MC, Traynor M, Bruce JL, Bekker W, Laing GL, Aho JM, et al. Surgical Considerations for Pediatric Snake Bites in Low‐ and Middle‐Income Countries. World j surg. 2019;43: 1636–1643. doi:[10.1007/s00268-019-04953-9](https://doi.org/10.1007/s00268-019-04953-9)
7. Ndu, I., Edelu, B., & Ekwochi, U. (2018). Snakebites in a Nigerian children Population: A 5-year review. *Sahel Medical Journal*, *21*(4), 204. <https://doi.org/10.4103/smj.smj_18_18>
8. Iliyasu, G., Dayyab, F. M., Michael, G. C., Hamza, M., Habib, M. A., Gutiérrez, J. M., & Habib, A. G. (2023). Case fatality rate and burden of snakebite envenoming in children – A systematic review and meta-analysis. Toxicon, 234, 107299. <https://doi.org/10.1016/j.toxicon.2023.107299>
9. Einterz EM, Bates ME. Snakebite in northern Cameroon: 134 victims of bites by the saw-scaled or carpet viper, Echis ocellatus. Trans R Soc Trop Med Hyg. 2003;97: 693–696. doi:[10.1016/s0035-9203(03)80105-0](https://doi.org/10.1016/s0035-9203(03)80105-0)
10. Buitendag J, Variawa S, Wood D, Oosthuizen G. An analysis of paediatric snakebites in north-eastern South Africa. S Afr j surg. 2021;59. doi:[10.17159/2078-5151/2021/v59n3a3500](https://doi.org/10.17159/2078-5151/2021/v59n3a3500)
11. Steegemans I, Sisay K, Nshimiyimana E, Gebrewold G, Piening T, Menberu Tessema E, et al. Treatment outcomes among snakebite patients in north-west Ethiopia—A retrospective analysis. Ainsworth SR, editor. PLoS Negl Trop Dis. 2022;16: e0010148. doi:[10.1371/journal.pntd.0010148](https://doi.org/10.1371/journal.pntd.0010148)
12. Nduagubam, O. C., Chime, O. H., Ndu, I. K., Bisi-Onyemaechi, A., Eke, C. B., Amadi, O. F., & Igbokwe, O. O. (2020). Snakebite in children in Nigeria: A comparison of the first aid treatment measures with the world health organization’s guidelines for management of snakebite in Africa. *Annals of African Medicine*, *19*(3), 182–187. <https://doi.org/10.4103/aam.aam_38_19>
13. Sloan, D. J., Dedicoat, M. J., & Lalloo, D. G. (2007). Healthcare‐seeking behaviour and use of traditional healers after snakebite in Hlabisa sub‐district, KwaZulu Natal. Tropical Medicine & International Health, 12(11), 1386–1390. <https://doi.org/10.1111/j.1365-3156.2007.01924.x>
14. Variawa S, Buitendag J, Marais R, Wood D, Oosthuizen G. Prospective review of cytotoxic snakebite envenomation in a paediatric population. Toxicon. 2021;190: 73–78. doi:[10.1016/j.toxicon.2020.12.009](https://doi.org/10.1016/j.toxicon.2020.12.009)
15. Wood, D., Sartorius, B., & Hift, R. (2016a). Classifying snakebite in South Africa: Validating a scoring system. *South African Medical Journal*, *107*(1), 46. <https://doi.org/10.7196/SAMJ.2017.v107i1.11361>
16. Wood, D., Sartorius, B., & Hift, R. (2016b). Snakebite in north-eastern South Africa: Clinical characteristics and risks for severity. *South African Family Practice*, *58*(2), 62–67. <https://doi.org/10.1080/20786190.2015.1120934>
17. Wood, D., Sartorius, B., & Hift, R. (2016). Classifying snakebite in South Africa: Validating a scoring system. *South African Medical Journal*, *107*(1), 46. <https://doi.org/10.7196/SAMJ.2017.v107i1.11361>
18. Wood D, Webb C, DeMeyer J. Severe snakebites in northern KwaZulu-Natal: treatment modalities and outcomes. S Afr Med J. 2009;99: 814–818.
