# Supplementary material for: Pediatric snakebite in Sub-Saharan Africa: Clinical predictors, outcomes, and gaps in care—A systematic review
Source: PLoS Negl Trop Dis. 2026 Feb 19;20(2):e0013450. doi: 10.1371/journal.pntd.0013450 (PMC12945311; doi:10.1371/journal.pntd.0013450)
Supplement: S4 Table — Country-level summary of antivenom availability and use, including reported products and supporting references from included studies. (DOCX) [file pntd.0013450.s004.docx]

# **S4_Table Antivenom Use, Products and References by Country**

| Country | Antivenom Product(s) | Source/Supplier | Study References | Notes |
| --- | --- | --- | --- | --- |
| South Africa | SAIMR Polyvalent | SAIMR/South African Vaccine Producers | [1-4] | Used in most cases; reserved for severe. |
| Nigeria | Echitab-Plus, EchiTAb G, VINS Polyvalent | EchiTAb Consortium, VINS Bioproducts, ICP Costa Rica | [5-7] | Echitab/ICP is most common; sometimes VINS. |
| Kenya | Inoserp Pan-Africa, Polyvalent (unspecified) | Inosan Biopharma, possibly SAIMR | [8] | Product type is inconsistently reported. |
| Gambia | Not specified (likely Indian Polyvalent or Inoserp) | Not specified | [9] | Product name not stated. |
| Ethiopia | Not available/Not administered |  | [10, 11] | Antivenom not available in included studies. |
| Cameroon | Not available/Not administered |  | [12] | No children received antivenom. |

**References**

1. Wood, D., Sartorius, B., & Hift, R. (2016a). Classifying snakebite in South Africa: Validating a scoring system. *South African Medical Journal*, *107*(1), 46. <https://doi.org/10.7196/SAMJ.2017.v107i1.11361>
2. Wood, D., Sartorius, B., & Hift, R. (2016b). Snakebite in north-eastern South Africa: Clinical characteristics and risks for severity. *South African Family Practice*, *58*(2), 62–67. <https://doi.org/10.1080/20786190.2015.1120934>
3. Wood, D., Webb, C., & DeMeyer, J. (2009). Severe snakebites in northern KwaZulu-Natal: Treatment modalities and outcomes. South African Medical Journal = Suid-Afrikaanse Tydskrif Vir Geneeskunde, 99(11), 814–818.
4. Hardcastle, T., Engelbrecht, A., Lalloo, V., Bell, C., & Toubkin, M. (2023). Approach to the diagnosis and management of snakebite envenomation in south africa in humans.: The hospital phase – emergency unit general principles. *South African Medical Journal*, 12–18. <https://doi.org/10.7196/SAMJ.2023.v113i6.1037>
5. Abouyannis, M., Boga, M., Amadi, D., Ouma, N., Nyaguara, A., Mturi, N., Berkley, J. A., Adetifa, I. M., Casewell, N. R., Lalloo, D. G., & Hamaluba, M. (2023). A long-term observational study of paediatric snakebite in Kilifi County, south-east Kenya. *PLOS Neglected Tropical Diseases*, *17*(7), e0010987. <https://doi.org/10.1371/journal.pntd.0010987>
6. Ndu, I., Edelu, B., & Ekwochi, U. (2018). Snakebites in a Nigerian children Population: A 5-year review. *Sahel Medical Journal*, *21*(4), 204. <https://doi.org/10.4103/smj.smj_18_18>
7. Habib, A. G., & Abubakar, S. B. (2011). Factors affecting snakebite mortality in north-eastern Nigeria. *International Health*, *3*(1), 50–55. <https://doi.org/10.1016/j.inhe.2010.08.001>
8. Abouyannis, M., Boga, M., Amadi, D., Ouma, N., Nyaguara, A., Mturi, N., Berkley, J. A., Adetifa, I. M., Casewell, N. R., Lalloo, D. G., & Hamaluba, M. (2023). A long-term observational study of paediatric snakebite in Kilifi County, south-east Kenya. *PLOS Neglected Tropical Diseases*, *17*(7), e0010987. <https://doi.org/10.1371/journal.pntd.0010987>
9. Habib, A. G., Kuznik, A., Hamza, M., Abdullahi, M. I., Chedi, B. A., Chippaux, J.-P., & Warrell, D. A. (2015). Snakebite is under appreciated: Appraisal of burden from west africa. *PLOS Neglected Tropical Diseases*, *9*(9), e0004088. <https://doi.org/10.1371/journal.pntd.0004088>
10. Abdullahi, A., Yusuf, N., Debella, A., Eyeberu, A., Deressa, A., Bekele, H., Ketema, I., Abdulahi, I. M., & Weldegebreal, F. (2022). Seasonal variation, treatment outcome, and its associated factors among the snakebite patients in Somali region, Ethiopia. *Frontiers in Public Health*, *10*, 901414. <https://doi.org/10.3389/fpubh.2022.901414>
11. Steegemans, I., Sisay, K., Nshimiyimana, E., Gebrewold, G., Piening, T., Menberu Tessema, E., Sahelie, B., Alcoba, G., Gebretsadik, F. S., Essink, D., Collin, S., Lucero, E., & Ritmeijer, K. (2022). Treatment outcomes among snakebite patients in north-west Ethiopia—A retrospective analysis. *PLOS Neglected Tropical Diseases*, *16*(2), e0010148. <https://doi.org/10.1371/journal.pntd.0010148>
12. Chippaux, J.-P. (2017). Snakebite envenomation turns again into a neglected tropical disease! *Journal of Venomous Animals and Toxins Including Tropical Diseases*, *23*(1), 38. <https://doi.org/10.1186/s40409-017-0127-6>
