# Supplementary material for: Pediatric snakebite in Sub-Saharan Africa: Clinical predictors, outcomes, and gaps in care—A systematic review
Source: PLoS Negl Trop Dis. 2026 Feb 19;20(2):e0013450. doi: 10.1371/journal.pntd.0013450 (PMC12945311; doi:10.1371/journal.pntd.0013450)
Supplement: S5 Table — Summary of country-level epidemiologic patterns and clinical outcomes, including mortality, amputation, permanent disability, median hospital stay, and key prognostic indicators reported across included studies. (DOCX) [file pntd.0013450.s005.docx]

## **Table S5. Country-level pediatric snakebite epidemiology, clinical outcomes, and prognostic indicators in SSA**

| Country | Mortality (%) | Amputation (%) | Permanent disability (%) | Median hospital stays (days) | Antivenom access | Key prognostic indicators / drivers of poor outcomes | Dominant syndromic pattern / species notes |
| --- | --- | --- | --- | --- | --- | --- | --- |
| South Africa  [1-3] | <1 | 1–2 | Rare | 4–7 | Yes | Better outcomes associated with timely presentation, antivenom availability, and access to surgical care | Cytotoxic/haematotoxic syndromes predominate (viperid species); neurotoxic syndromes generally not confirmed |
| Kenya [4] | <1 | 0.5 | Rare | NR | Yes | Improved outcomes linked to timely access to antivenom and supportive care | Predominantly cytotoxic/haematotoxic patterns; neurotoxic syndromes may be under-recognized |
| Gambia [5] | 14 | NR | NR | NR | Limited | Worse outcomes linked to limited antivenom availability and delayed presentation | Cytotoxic/haematotoxic patterns reported; limited species confirmation in some reports |
| Cameroon [6] | 36 | NR | NR | NR | Limited | High mortality associated with limited anti-venom access, delayed care, and systemic complications | Predominantly cytotoxic/haematotoxic syndromes (viperid species) |
| Ethiopia [7,8] | 4.7–11 | 6 | NR | NR | No/limited | Poor outcomes associated with delayed presentation, severe local swelling/necrosis, and systemic complications | Cytotoxic/haematotoxic syndromes common; neurotoxic syndromes not consistently reported |
| Nigeria [9-11] | 3–7.7 | 1.7 | NR | NR | Limited | Worse outcomes linked to delayed presentation, inconsistent access to antivenom, and severe local/systemic effects | Cytotoxic/haematotoxic patterns common; neurotoxic syndromes may be under-recognized |

***Abbreviations****: AKI, acute kidney injury; NR, not reported; SBE, snakebite envenomation; SI, Supporting Information.*

**References**

1. Wood D, Sartorius B, Hift R. Classifying snakebite in South Africa: Validating a scoring system. S Afr Med J. 2016;107: 46. doi:10.7196/SAMJ.2017.v107i1.11361
2. Wood, D., Sartorius, B., & Hift, R. (2016). Snakebite in north-eastern South Africa: Clinical characteristics and risks for severity. *South African Family Practice*, *58*(2), 62–67. <https://doi.org/10.1080/20786190.2015.1120934>
3. Wood, D., Webb, C., & DeMeyer, J. (2009). Severe snakebites in northern KwaZulu-Natal: Treatment modalities and outcomes. *South African Medical Journal = Suid-Afrikaanse Tydskrif Vir Geneeskunde*, *99*(11), 814–818.
4. Abouyannis, M., Boga, M., Amadi, D., Ouma, N., Nyaguara, A., Mturi, N., Berkley, J. A., Adetifa, I. M., Casewell, N. R., Lalloo, D. G., & Hamaluba, M. (2023). A long-term observational study of paediatric snakebite in Kilifi County, south-east Kenya. *PLOS Neglected Tropical Diseases*, *17*(7), e0010987. <https://doi.org/10.1371/journal.pntd.0010987>
5. Habib, A. G., Kuznik, A., Hamza, M., Abdullahi, M. I., Chedi, B. A., Chippaux, J.-P., & Warrell, D. A. (2015). Snakebite is under appreciated: Appraisal of burden from west africa. *PLOS Neglected Tropical Diseases*, *9*(9), e0004088. <https://doi.org/10.1371/journal.pntd.0004088>
6. Einterz EM, Bates ME. Snakebite in northern Cameroon: 134 victims of bites by the saw-scaled or carpet viper, Echis ocellatus. Trans R Soc Trop Med Hyg. 2003;97: 693–696. doi:[10.1016/s0035-9203(03)80105-0](https://doi.org/10.1016/s0035-9203(03)80105-0)
7. Abdullahi, A., Yusuf, N., Debella, A., Eyeberu, A., Deressa, A., Bekele, H., Ketema, I., Abdulahi, I. M., & Weldegebreal, F. (2022). Seasonal variation, treatment outcome, and its associated factors among the snakebite patients in Somali region, Ethiopia. *Frontiers in Public Health*, *10*, 901414. <https://doi.org/10.3389/fpubh.2022.901414>
8. Steegemans, I., Sisay, K., Nshimiyimana, E., Gebrewold, G., Piening, T., Menberu Tessema, E., Sahelie, B., Alcoba, G., Gebretsadik, F. S., Essink, D., Collin, S., Lucero, E., & Ritmeijer, K. (2022). Treatment outcomes among snakebite patients in north-west Ethiopia—A retrospective analysis. *PLOS Neglected Tropical Diseases*, *16*(2), e0010148. <https://doi.org/10.1371/journal.pntd.0010148>
9. Habib, A. G., & Abubakar, S. B. (2011). Factors affecting snakebite mortality in north-eastern Nigeria. *International Health*, *3*(1), 50–55. <https://doi.org/10.1016/j.inhe.2010.08.001>
10. Ndu, I., Edelu, B., & Ekwochi, U. (2018). Snakebites in a Nigerian children Population: A 5-year review. *Sahel Medical Journal*, *21*(4), 204. <https://doi.org/10.4103/smj.smj_18_18>
11. Nduagubam, O. C., Chime, O. H., Ndu, I. K., Bisi-Onyemaechi, A., Eke, C. B., Amadi, O. F., & Igbokwe, O. O. (2020). Snakebite in children in Nigeria: A comparison of the first aid treatment measures with the world health organization’s guidelines for management of snakebite in Africa. *Annals of African Medicine*, *19*(3), 182–187. <https://doi.org/10.4103/aam.aam_38_19>
