# Supplementary material for: Pediatric snakebite in Sub-Saharan Africa: Clinical predictors, outcomes, and gaps in care—A systematic review
Source: PLoS Negl Trop Dis. 2026 Feb 19;20(2):e0013450. doi: 10.1371/journal.pntd.0013450 (PMC12945311; doi:10.1371/journal.pntd.0013450)
Supplement: S6 Table — Summary of clinical and laboratory predictors of severe outcomes, including odds ratios and 95% confidence intervals, as presented in the narrative synthesis. (DOCX) [file pntd.0013450.s006.docx]

## **S6_Table Predictors of Severe Outcomes in Pediatric Snakebite in Sub-Saharan Africa**

| Ref. No | Predictor (risk factor) | Odds Ratio (OR) | 95% Confidence Interval (CI) | Interpretation |
| --- | --- | --- | --- | --- |
| [1] | Rural residence | 2.9 | 1.4–5.7 | Children living in rural areas had higher odds of severe outcomes. |
| [5,6] | Traditional first aid | 2.8 | 1.5–5.1 | Use of traditional first aid was associated with increased severity. |
| [3,6] | Elevated INR | 3.4 | 1.6–7.0 | Coagulopathy was strongly associated with severe outcomes. |
| [4,9] | Low hemoglobin | 2.0 | 1.1–3.8 | Anemia was associated with higher odds of severe outcomes. |
| [9,10] | Leukocytosis | 2.1 | 1.0–4.0 | Elevated white cell count was associated with increased severity. |
| [4, 8, 10] | Severe swelling/blistering | 3.8 | 2.1–6.9 | Severe local tissue injury was associated with worse outcomes. |
| [7] | Age <10 years | 2.3 | 1.1–4.6 | Children under 10 years had higher odds of severe outcomes. |
| [1,2,5,6,7] | Delayed presentation (>6 hours) | 5.1 | 3.1–8.7 | Strongest predictor: delayed presentation markedly increased the odds of severe outcomes. |

***Legend:*** *Odds ratios (OR) and 95% confidence intervals (CI) represent associations between clinical/laboratory predictors and severe outcomes in pediatric snakebite.*

**References**

1. Abdullahi, A., Yusuf, N., Debella, A., Eyeberu, A., Deressa, A., Bekele, H., Ketema, I., Abdulahi, I. M., & Weldegebreal, F. (2022). Seasonal variation, treatment outcome, and its associated factors among the snakebite patients in Somali region, Ethiopia. *Frontiers in Public Health*, *10*, 901414. <https://doi.org/10.3389/fpubh.2022.901414>
2. Buitendag J, Variawa S, Wood D, Oosthuizen G. An analysis of paediatric snakebites in north-eastern South Africa. S Afr j surg. 2021;59. doi:[10.17159/2078-5151/2021/v59n3a3500](https://doi.org/10.17159/2078-5151/2021/v59n3a3500)
3. Hadley GP, McGarr P, Mars M. The role of thromboelastography in the management of children with snake-bite in southern Africa. Transactions of the Royal Society of Tropical Medicine and Hygiene. 1999;93: 177–179. doi:[10.1016/S0035-9203(99)90300-0](https://doi.org/10.1016/S0035-9203(99)90300-0)
4. Hernandez MC, Traynor M, Bruce JL, Bekker W, Laing GL, Aho JM, et al. Surgical Considerations for Pediatric Snake Bites in Low‐ and Middle‐Income Countries. World j surg. 2019;43: 1636–1643. doi:[10.1007/s00268-019-04953-9](https://doi.org/10.1007/s00268-019-04953-9)
5. Ndu, I., Edelu, B., & Ekwochi, U. (2018). Snakebites in a Nigerian children Population: A 5-year review. *Sahel Medical Journal*, *21*(4), 204. <https://doi.org/10.4103/smj.smj_18_18>
6. Iliyasu, G., Dayyab, F. M., Michael, G. C., Hamza, M., Habib, M. A., Gutiérrez, J. M., & Habib, A. G. (2023). Case fatality rate and burden of snakebite envenoming in children – A systematic review and meta-analysis. Toxicon, 234, 107299. <https://doi.org/10.1016/j.toxicon.2023.107299>
7. Nduagubam, O. C., Chime, O. H., Ndu, I. K., Bisi-Onyemaechi, A., Eke, C. B., Amadi, O. F., & Igbokwe, O. O. (2020). Snakebite in children in Nigeria: A comparison of the first aid treatment measures with the world health organization’s guidelines for management of snakebite in Africa. *Annals of African Medicine*, *19*(3), 182–187. <https://doi.org/10.4103/aam.aam_38_19>
8. Variawa S, Buitendag J, Marais R, Wood D, Oosthuizen G. Prospective review of cytotoxic snakebite envenomation in a paediatric population. Toxicon. 2021;190: 73–78. doi:[10.1016/j.toxicon.2020.12.009](https://doi.org/10.1016/j.toxicon.2020.12.009)
9. Wood, D., Sartorius, B., & Hift, R. (2016a). Classifying snakebite in South Africa: Validating a scoring system. *South African Medical Journal*, *107*(1), 46. <https://doi.org/10.7196/SAMJ.2017.v107i1.11361>
10. Abouyannis, M., Boga, M., Amadi, D., Ouma, N., Nyaguara, A., Mturi, N., Berkley, J. A., Adetifa, I. M., Casewell, N. R., Lalloo, D. G., & Hamaluba, M. (2023). A long-term observational study of paediatric snakebite in Kilifi County, south-east Kenya. PLOS Neglected Tropical Diseases, 17(7), e0010987. <https://doi.org/10.1371/journal.pntd.0010987>
