# Supplementary material for: Pediatric snakebite in Sub-Saharan Africa: Clinical predictors, outcomes, and gaps in care—A systematic review
Source: PLoS Negl Trop Dis. 2026 Feb 19;20(2):e0013450. doi: 10.1371/journal.pntd.0013450 (PMC12945311; doi:10.1371/journal.pntd.0013450)
Supplement: Attachment — Submitted filename: Response to editoral and reviewers comments_final_V3.docx [file pntd.0013450.s008.docx]

**PNTD-D-25-01371**

**Pediatric Snakebite in Sub-Saharan Africa: Clinical Predictors, Outcomes, and Gaps in Care—A Systematic Review**

We thank the editors and reviewers for their valuable and constructive comments. We have carefully revised the manuscript as suggested. Below, we provide a point-by-point response to the reviewers’ comments.

Journal Requirements

Comment#1: Please ensure that the CRediT author contributions listed for every co-author are completed accurately and in full.

At this stage, the following Authors/Authors require contributions: Samuel Mayeden, Margit Wirth, Nisreen Agbaria, Masood Ali Shaikh, Peter Dambach, Michael Lowery Wilson, Olaf Horstick, Salvador Camacho, Hans-Jörg Lang, and Andreas Deckert.

Please ensure that the full contributions of each author are acknowledged in the "Add/Edit/Remove Authors" section of our submission form.

The list of CRediT author contributions may be found here: <https://journals.plos.org/plosntds/s/authorship#loc-author-contributions>

Response#1: Thank you very much for this comment. All CRediT author contributions have now been carefully reviewed and completed in full for all co-authors (Samuel Yao Mayeden, Margit Wirth, Nisreen Agbaria, Masood Ali Shaikh, Peter Dambach, Michael Lowery Wilson, Olaf Horstick, Salvador Camacho, Hans-Jörg Lang, and Andreas Deckert) within the Editorial Manager system. Each contribution has been verified to accurately reflect the respective roles and responsibilities of all authors in accordance with the PLOS CRediT taxonomy.

Comment#2: Please provide an Author Summary. This should appear in your manuscript between the Abstract (if applicable) and the Introduction, and should be 150-200 words long. The aim should be to make your findings accessible to a wide audience that includes both scientists and non-scientists.

Response#2: Thank you very much for this helpful suggestion. We have now added an Author summary section in accordance with PLOS NTD requirements. This section has been placed between the abstract and the introduction on page 2, lines 63–92 of the revised manuscript and is 188 words in length.

The summary provides a concise and accessible overview of the study’s scope, key findings, and implications for clinical practice and health policy, written to ensure comprehension by both scientific and non-specialist readers.

Comment#3: Please upload all main figures as separate Figure files in .tif or .eps format. For more information about how to convert and format your figure files please see our guidelines

Response#3: Thank you for this instruction. All nine figures have now been converted into .tif and .eps formats in accordance with PLOS NTD figure submission guidelines and have been uploaded as separate files within the submission system. Additionally, a consolidated .zip folder containing all formatted figure files has been provided for convenience.

Comment#4: Some material included in your submission may be copyrighted. According to PLOSu2019s copyright policy, authors who use figures or other material (e.g., graphics, clipart, maps) from another author or copyright holder must demonstrate or obtain permission to publish this material under the Creative Commons Attribution 4.0 International (CC BY 4.0) License used by PLOS journals. *Please respond directly to this email and provide any known details concerning your material's license terms and permissions required for reuse, even if you have not yet obtained copyright permissions or are unsure of your material's copyright compatibility.*

Once you have responded and addressed all other outstanding technical requirements, you may resubmit your manuscript within Editorial Manager.

Potential Copyright Issues:

- ***Figure 2.*** Please (a) provide a direct link to the base layer of the map (i.e., the country or region border shape) and ensure this is also included in the figure legend; and (b) provide a link to the terms of use / license information for the base layer image or shapefile. We cannot publish proprietary or copyrighted maps (e.g. Google Maps, Mapquest) and the terms of use for your map base layer must be compatible with our CC BY 4.0 license.

Response#4: Thank you for highlighting this important point. Figure 2 has been revised to include full citation of the basemap source. The following text has now been added to the figure legend on page 12, lines 384–386 of the revised manuscript:

*“Basemap source: Natural Earth (public domain data,* [*https://www.naturalearthdata.com*](https://www.naturalearthdata.com)*). Map data freely available under CC0 1.0 Public Domain license.”*

This ensures full compliance with PLOS open-access and copyright requirements.

**Reviewer’s Comments**

Reviewer#1

Comment #1: This is a useful systematic review; however the context is not given in the Discussion. The differences between species across the region and therefore the need for antivenom are very different, for examples SA does not have Echis, which is common in central SSA, for which there is a specific antivenom, while most SA bites are not AV requiring. The lack of "neurotoxic" bites mentioned in the discussion is inaccurate as a number of the papers from Wood et al. include neurotoxic cases. See S Afr Med J 2009; 99: 814-818. Also see S Afr Med J 2017;107(12):1082-1085. DOI:10.7196/SAMJ. 2017.v107i12.12628 which focusses on cytotoxic bites.

Response #1: Thank you very much for this valuable comment. We expanded the Discussion section to clarify regional ecological differences in snake epidemiological distribution and their implications for antivenom use and clinical pattern recognition. We addressed this, on page 16, lines 414–421 of our revised manuscript:

*“Regional variation in dominant snake species also influences clinical manifestations and antivenom efficacy. Cytotoxic and haematotoxic syndromes, largely due to viperid species (Bitis and Echis), predominate across SSA, though rear-fanged colubrids may also cause haematotoxicity. Elapid snakes (e.g., Naja, Dendroaspis, Hemachatus) are responsible for neurotoxic syndromes, often with overlapping cytotoxicity in spitting cobras. Differences in regional snake ecology must be considered in syndrome-specific clinical training and antivenom selection.”*

Additionally, we acknowledged the presence of neurotoxic envenoming in the region, we added the following clarification in the Discussion section on page 17, lines 431–435:

*“Although none of the included studies reported confirmed neurotoxic syndromes, this likely reflects underreporting or diagnostic limitations rather than a true absence. Neurotoxic envenoming from Naja annulifera and Dendroaspis polylepis is well documented in southern Africa [37,23]. The lack of pediatric reports may reflect ecological variation or pre-hospital mortality due to rapid paralysis before reaching care”*

Reviewer #2:

Comment #1: Primary Causative Snake Species: While we do not advocate using snake species as the primary criterion for treatment or management, but identifying the predominant snake species responsible for envenomation would hold significant public health value for local prevention and awareness campaigns.

Response #1: Thank you for this helpful comment. We agree that identifying the predominant snake species responsible for envenomation is important for guiding prevention and public awareness, even though clinical management should remain based on syndrome presentation.

To address this, we added the following text in the Discussion section as suggested on page 16, lines 414–421 of the revised manuscript:

*“Regional variation in dominant snake species also influences clinical manifestations and antivenom efficacy. Cytotoxic and haematotoxic syndromes, largely due to viperid species (Bitis and Echis), predominate across SSA, though rear-fanged colubrids may also cause haematotoxicity. Elapid snakes (Naja, Dendroaspis, and Hemachatus) are responsible for neurotoxic syndromes, often with overlapping cytotoxicity in spitting cobras. Differences in regional snake ecology must be considered in syndrome-specific clinical training and antivenom selection.”*

We further elaborated on the relevance of these ecological and clinical distinctions for patient management and health system planning on page 16, lines 422–425:

*“These ecological and clinical differences are essential for designing pragmatic, syndrome-specific training and improving the selection and distribution of antivenoms”*

Additionally, to reinforce alignment with WHO policy guidance on antivenom suitability and coverage across species distributions, we added the following clarification on page 16, lines 426–430:

*“In line with WHO guidance, snake distribution patterns inform recommended antivenom use; however, not all medically important African species are adequately covered by currently available antivenoms. This highlights the need for regionally appropriate antivenom development, improved supply chain management systems, and strengthened diagnostic and treatment capacity.”*

Comment #2: Mortality and Disability Analysis: If feasible, I suggest supplementing the review with a more detailed discussion or synthesis of the causes of mortality and long-term disability, where available data permits.

Response #2: Thank you for this valuable suggestion. We have expanded the discussion to provide a more detailed synthesis of the causes of mortality and long-term disability. The added extracted additional data and added to the manuscript the text below in Result, Discussion and Conclusion sections on page 18, lines 457–460:

*“Across studies, pediatric mortality was approximately 6.6%, most commonly resulting from haemodynamic instability (e.g., septic or haemorrhagic shock) and respiratory failure. Permanent disability occurred in about 4% of survivors, primarily due to necrosis-related amputations or contractures associated with delayed presentation.”*

To ensure transparency, we have also referred to these quantitative patterns within the Results section and linked them directly to the supporting dataset in Table S7 (Supplementary Information).

Furthermore, this interpretation is reiterated within the Conclusion section on pages 19–20, lines 491–494, where we added:

*“These outcomes are largely preventable through timely access to supportive care, appropriate use of WHO-recommended antivenoms, and comprehensive rehabilitation services.”*

Comment #3: Age: The statement that “The affected children were predominantly under 12 years of age” is somewhat broad. Could you specify the exact age range, median, or average age of snakebite? Thank you very much! Rongde Lai (Emergency Department, the First Affiliated Hospital of Guangzhou Medical University) 2025-9-22

Response #3: Thank you very much for this helpful observation. We have clarified the age information to provide greater precision. We have added to the manuscript in the Results section (Section 3.4, page 13, lines 349–352) as below:

*“The pooled median age was 9 years (IQR 6–12; range 1–17 years), and males accounted for 68% of all pediatric cases.”*

Reviewer #3

Comment #1: *Abstract* This should be rewritten concerning the Methods. Methods. ….published from……..to ….. Need to include the duration. Age should be defined what languages used? Databases and keywords should be included.

Response #1: Thank you very much for this helpful feedback. We have revised the Abstract to clearly describe the methodology, including the study period, databases searched, and inclusion criteria. The revised version, on page 1, lines 17–32, now includes the following:

“*We systematically searched PubMed, SafetyLit, African Journals Online, CINAHL, and Google Scholar for studies published up to October 2024 on pediatric snakebite in sub-Saharan Africa. Eligible studies included children under 18 years of age. Data on epidemiology, clinical features, management, and outcomes were extracted and synthesized*.”

Additionally, we modified the concluding component of the Abstract to ensure that the methodological elements are clearly summarized:

“*The abstract also summarizes the search strategy and key outcomes to ensure transparency and methodological clarity*.”

Comment #2: Methods: What is the basis of selecting only 5 databases? What languages were used? This should be included in to the inclusion/exclusion criteria. Inclusion/exclusion criteria. How did the authors confirm that selected papers are peer-reviewed or not?

Response #2: Thank you for this valuable comment. The rationale for selecting five databases has been clarified in the manuscript. In the Methods on section 2.1, page 6, lines 169–176, we included the following explanation:

*“We systematically searched five databases; PubMed, Scopus, Embase, Web of Science, and African Journals Online (AJOL) to ensure comprehensive coverage of both international and region-specific literature relevant to pediatric snakebite in SSA.”*

In addition, the Eligibility subsection has been revised to explicitly state both the language approach and peer-review verification. In Section 2.2 on page 7, lines 187–193, the following clarification has been added:

*“No language restrictions were imposed during the search. Only articles published in peer-reviewed journals were included, while conference abstracts, theses, preprints, and non-peer-reviewed materials were excluded.”*

Comment #3: Introduction. It is better to include contents of some landmark paed snakebite papers other than in SSA.…….definitive care defined as defined as timely…..

Response #3: Thank you for this helpful suggestion. We have expanded our Introduction section to include landmark pediatric snakebite publications beyond SSA. The following were added to the Introduction section on pages 5–6.

*“Landmark pediatric snakebite studies from Asia and Latin America have similarly identified high risk among children for severe envenoming complications, delays to care, and inequitable access to antivenom [5–8]. These global findings highlight that pediatric snakebite is not confined to SSA but represents a widespread pediatric vulnerability across snakebite-endemic regions.”*

*…and delays in receiving definitive care, defined as timely access to antivenom administration and supportive critical care within a health facility equipped to manage envenoming-related complications– [8,10].*

*Comparing patterns across global regions provides important context for understanding fundamental clinical risks in pediatric snakebite and underscores the need for regionally grounded treatment strategies [5–7,11].*

*Most published literature focuses on broad epidemiology or clinical manifestations, and only a few studies systematically examine which clinical, demographic, or health system-related factors are associated with poor outcomes among children [9,10].*

Comment #4: Figure 3, 4, 5 and 7. The legend should be placed below the figure.

Response #4: Thank you for this helpful observation. The legends for Figures 3, 4, 5, and 7 have now been repositioned below each respective figure in accordance with the journal’s formatting requirements. These adjustments appear in the revised manuscript on pages 11–15. Additionally, we reviewed all figure captions to ensure consistency in formatting, terminology, and clarity across all visual elements in the manuscript.

Comment #5: Results. How many known snakebites and unknown snakebites? In known snakebites, classify them according to the snake species. The systemic manifestations have been quantified as 3%, 4% and 2% in major SSA countries.

What are those manifestations? Include their numbers. This should be discussed in the Discussion. ……A striking finding was also the absence of reported neurotoxic syndromes among the included studies. In practical point of view, this cannot happen because in this region, there are neurotoxic snakes. Please clarify this.

Response #5: Thank you for these detailed comments. The Results section 3.6, pages 14–16, lines 369–410) now explicitly describes the classification of snakebites, distinguishing between known and unknown species where additional data were extracted. Specifically, studies that identified causative species were summarized according to the dominant cytotoxic and haematotoxic viperid species (*Echis*, *Bitis*) and neurotoxic elapid species (*Naja*, *Dendroaspis*).

We also clarified the systemic complications reported across SSA cohorts, namely:

- coagulopathy: 3%,
- acute kidney injury (AKI): 4%, and
- shock: 2%,

as presented in the Results. Importantly, we noted also that the reporting of clinical snakebite syndromes was inconsistent across studies. In some cases, complications may have resulted from direct venom effects, while in others they were associated with secondary consequences, such as superinfected necrosis leading to sepsis.

We further expanded this in the Discussion section to ensure that the absence of reported neurotoxic syndromes in the included studies is not misinterpreted as evidence of their true absence. In the revised manuscript (pages 18–19, lines 458–482), we explained that this finding likely reflects (i) diagnostic and clinical recognition limitations, (ii) regional ecological variation in the distribution of neurotoxic snake species, and (iii) possible pre-hospital mortality due to rapidly progressive paralysis prior to reaching care.

To directly contextualize this, we additionally cited evidence from Wood et al. (S Afr Med J 2009; 2017), documenting neurotoxic pediatric envenoming from *Naja annulifera* and *Dendroaspis polylepis* in Southern Africa. These additions acknowledge that neurotoxic presentations do occur in the region and that their absence in the included pediatric studies should be interpreted cautiously.

Furthermore, to ensure transparency, supporting data on reported clinical features and outcomes are now cross-referenced in Table S4 (Supporting Information). This revision strengthens the Discussion and aligns it with the reviewer’s recommendation.

Comment #6: Discussion. It is better to compare SSA results with results of other parts of the world. Other than that, the discussion is OK.

Response #6: Thank you very much for this helpful suggestion. In response, we have expanded the Discussion to include a broader international comparative analysis. This now situates the findings from SSA within the wider context of pediatric snakebite outcomes observed in South/Southeast Asia and Latin America.

Specifically, we added the following comparative text to the Discussion on pages 19–21, lines 483–505):

*“To provide an international context, our findings in SSA can be compared with trends observed in other regions where pediatric snakebite burden is high. In South and Southeast Asia, for example, mortality among children treated in hospital settings typically ranges from 2–5%, substantially lower than the average mortality observed in SSA (6–8%), likely reflecting earlier presentation and broader antivenom availability. In Latin America, despite different snake fauna dominated by Bothrops and Crotalus species, necrosis-related disability patterns among survivor’s mirror those seen in SSA, with amputation, limb deformity, and contractures representing major contributors to long-term morbidity. These cross-regional comparisons underscore the importance of contextualizing SSA outcomes within global experiences and suggest that improvements in pre-hospital care, antivenom access, and early clinical recognition as implemented in parts of Asia may offer feasible models to reduce mortality in SSA pediatric populations.”*

In addition, to reinforce global context, we referenced landmark international evidence in the Discussion, citing Warrell & Williams (2023) and Longbottom et al. (2018), which highlight disparities in antivenom distribution, treatment accessibility, and health system preparedness worldwide.

These additions strengthen the global relevance of our analysis and ensure that the Discussion reflects international patterns while maintaining focus on the unique clinical and public health challenges in SSA.

We also appreciate the reviewer’s suggestion, which significantly enhanced the clarity and broader applicability of our findings.
