# Supplementary material for: Pediatric snakebite in Sub-Saharan Africa: Clinical predictors, outcomes, and gaps in care—A systematic review
Source: PLoS Negl Trop Dis. 2026 Feb 19;20(2):e0013450. doi: 10.1371/journal.pntd.0013450 (PMC12945311; doi:10.1371/journal.pntd.0013450)
Supplement: Attachment — Submitted filename: Response to reviewers and editor.docx [file pntd.0013450.s009.docx]

**RESPONSE TO REVIEWERS & EDITOR**

We would like to thank the reviewers and editor for your careful evaluation of our manuscript entitled **“Pediatric Snakebite in Sub-Saharan Africa: Clinical Predictors, Outcomes, and Gaps in Care—A Systematic Review”** (Manuscript ID: **PNTD-D-25-01371**). We greatly appreciate the constructive comments and suggestions, which have helped us improve the clarity, rigor, and overall quality of our manuscript. We have revised the manuscript accordingly and addressed all points raised. For ease of review, we provide a point-by-point response in the table below and have indicated where changes have been made in the revised manuscript (with page and line references), and we have included tracked changes to highlight all modifications.

| **Comment** | **Response** |
| --- | --- |
| **Journal Requirements** | |
| 1) Please upload all main figures as separate Figure files in .tif or .eps format. For more information about how to convert and format your figure files please see our guidelines:  <https://journals.plos.org/plosntds/s/figures> | We thank the editorial office for this observation. All main figures have now been uploaded as separate figure files in **.tif** format, in full compliance with the PLOS Neglected Tropical Diseases figure preparation guidelines. |
| 2) We have noticed that you have uploaded Supporting Information files, but you have not included a list of legends. Please add a full list of legends for your Supporting Information files after the references list. | We thank the editorial office for this observation. A complete “Legends for Supporting Information” section has now been added after the references in the revised manuscript, and have provided full legends for all Supporting Information files (Tables S1–S6) as requested. |
| 3) We notice that your supplementary figures are uploaded with the file type 'Table'. Please amend the file type to 'Supporting Information'. Please ensure that each Supporting Information file has a legend listed in the manuscript after the references list. | We thank the editorial office for this observation. All supplementary figures have been reclassified under the correct file type (**“Supporting Information”**). Each Supporting Information file is appropriately labeled and has a corresponding legend listed in the manuscript after the references. |
| **Reviewers' Comments:** | |
| Reviewer's Responses to Questions  **Key Review Criteria Required for Acceptance?** As you describe the new analyses required for acceptance, please consider the following: | |
| **Methods:**   - Are the objectives of the study clearly articulated with a clear testable hypothesis stated? - Is the study design appropriate to address the stated objectives? - Is the population clearly described and appropriate for the hypothesis being tested? - Is the sample size sufficient to ensure adequate power to address the hypothesis being tested? - Were correct statistical analysis used to support conclusions? - Are there concerns about ethical or regulatory requirements being met? | |
| **Reviewer #1**: All changes acceptable | We thank reviewer for confirming that all methodological aspects of the study are acceptable. |
| **Reviewer #2**: - | No additional comments were provided |
| **Reviewer #3**: (No Response) | No response was received |
| **Results:**   - Does the analysis presented match the analysis plan? - Are the results clearly and completely presented? - Are the figures (Tables, Images) of sufficient quality for clarity? | |
| **Reviewer #1**: All changes acceptable | We thank reviewer for confirming that the Results section is acceptable. |
| **Reviewer #2**: - | No additional comments were provided. |
| **Reviewer #3**: (No Response) | No response was provided. |
| **Conclusions:**   - Are the conclusions supported by the data presented? - Are the limitations of analysis clearly described? - Do the authors discuss how these data can be helpful to advance our understanding of the topic under study? - Is public health relevance addressed? | |
| **Reviewer #1**: All changes acceptable | We thank reviewer for confirming that the Conclusions section is acceptable. |
| **Reviewer #2**: - | No additional comments were provided. |
| **Reviewer #3**: (No Response) | No response was provided. |
| **Editorial and Data Presentation Modifications?**  Use this section for editorial suggestions as well as relatively minor modifications of existing data that would enhance clarity. If the only modifications needed are minor and/or editorial, you may wish to recommend “Minor Revision” or “Accept”. | |
| **Reviewer #1**: All changes acceptable | We thank Reviewer #1 for confirming that the editorial quality and data presentation are acceptable. |
| **Reviewer #2**: - | No additional comments were. |
| **Reviewer #3**: (No Response) | No response was provided. |
| **Summary and General Comments:** Use this section to provide overall comments, discuss strengths/weaknesses of the study, novelty, significance, general execution and scholarship. You may also include additional comments for the author, including concerns about dual publication, research ethics, or publication ethics. If requesting major revision, please articulate the new experiments that are needed. | |
| **Reviewer #1**: All changes acceptable | We thank Reviewer #1 for confirming that the editorial quality and data presentation are acceptable. |
| **Reviewer #2:** - | No comments were provided by Reviewer #2. |
| **Reviewer #3**: Pediatric Snakebite in Sub-Saharan Africa: Clinical Predictors, Outcomes, and Gaps in Care-A Systematic Review |  |
| Most of my queries have been addressed; however, a few revisions are still required. | We thank the reviewer for this important observation. We agree that the Results section should remain strictly descriptive, while interpretive statements should be presented in the Discussion. To strengthen clarity and ensure full consistency between the Results narrative and the visual summary (Figure 7), we consolidated the Supporting Information tables relevant to Section 3.8.  Specifically, Tables S5–S7 have been merged into a single Supporting Information table (Table S5), which now presents country-level pediatric snakebite epidemiology, clinical outcomes, and key prognostic indicators in one integrated summary aligned with Figure 7 (Panels A and B). The Results text in Section 3.8 has been revised accordingly, and all in-text citations have been updated to reflect the merged table numbering. |
| Line 172-176 If no language restrictions were applied, were there any articles published in languages other than English? If so, which languages were included, and how were their contents incorporated into the review? | We thank the reviewer for this important clarification. Although no language restrictions were applied during the database search, all studies that met the inclusion criteria and were included in the final review were published in English. Non-English records were screened at the title and abstract stage where an English abstract was available; however, studies without an English abstract or full text were excluded due to feasibility constraints. This clarification has been added to the methods section (**page 5; line 173–177**) of the revised manuscript:  *“However, all studies that met the inclusion criteria and included in the final review were published in English. Non-English records were screened at the title and abstract level where an English abstract was available; studies published without an English abstract or full text were excluded due to feasibility constraints.”* |
| **Results:** This section includes information that should be moved to the Discussion. Some examples are indicated below. Please correct these. Line 276-278  Importantly, compartment syndrome diagnosis should be approached cautiously in this context, as clinical assessment may overestimate the need for fasciotomy and intra-compartmental pressures are often not markedly elevated [9] | We thank the reviewer for this important observation. We agree that this statement is interpretive and is better placed in the Discussion section. Therefore, we removed this interpretive sentence from the Results section and relocated it to the Discussion section, where it is now presented in the appropriate context. The Results section has been revised to retain only descriptive findings, and the interpretive statement has been relocated to the discussion section **(page 16; line 387-390).**  *“Importantly, compartment syndrome diagnosis should be approached cautiously in this context, as reliance on clinical assessment alone may overestimate the need for fasciotomy, given that intra-compartmental pressures are often not markedly elevated [9].”* |
| Line 313-317 Among known snakebites, most were due to cytotoxic and haematotoxic viperid species (Echis, Bitis), consistent with regional epidemiology [25]. A smaller proportion of bites were attributed to neurotoxic elapids (Naja, Dendroaspis), although neurotoxic envenoming may be under-recognized in pediatric data due to diagnostic limitations and pre-hospital mortality [6,7,17]. | We thank the reviewer for this important observation. We agree that the statement regarding possible under-recognition of neurotoxic envenoming is interpretive and should be presented in the Discussion rather than the Results section. Therefore, we revised the Results section to retain only descriptive findings on the distribution of reported snake species and syndromic patterns and moved the interpretive statement to the discussion section where it is presented in the appropriate context; (**page 15-16; lines 355-359).**  *“Although no confirmed neurotoxic syndromes were reported among the pediatric cases in the included studies, this likely reflects diagnostic and reporting limitations, ecological variation in neurotoxic species distribution, and possible pre-hospital mortality due to rapidly progressive respiratory failure [6,7,17], rather than a true absence of neurotoxic envenoming in SSA [8,26]”.* |
| Line 328-330 …..most commonly resulting from haemodynamic instability (septic or haemorrhagic shock) and respiratory failure, consistent with recognized mortality mechanisms in pediatric envenoming [25,26]. | We thank the reviewer for this important observation. We agree that this sentence contains interpretive discussion and should not appear in the Results section. Therefore, we revised the Results to retain only descriptive findings and moved the interpretive statement to the discussion section, where it is now integrated to support interpretation of mortality mechanisms; (**page 16; lines 372-374).**  *“…substantially lower than the average mortality observed in SSA (6–8%), likely reflecting earlier presentation and broader antivenom availability [15,16,26].”* |
